# Anticancer activity of *Pupalia lappacea* on chronic myeloid leukemia K562 cells

**DOI:** 10.1186/2008-2231-20-86

**Published:** 2012-12-05

**Authors:** Alvala Ravi, Mallika Alvala, Venkatesh Sama, Arunasree M Kalle, Vamshi K Irlapati, B Madhava Reddy

**Affiliations:** 1 G.Pulla Reddy College of Pharmacy, Mehdipatnam, Hyderabad, 500 028, AP, India; 2 Birla Institute of Technology and Science, Pilani-Hyderabad Campus, Hyderabad, 500 078, AP, India; 3 Department of Animal Sciences, School of Life Sciences, University of Hyderabad, Hyderabad, 500046, India; 4 Institute of Life Sciences, University of Hyderabad Campus, Hyderabad, AP, 500 046, India

**Keywords:** Pupalia lappacea, Anticancer activity, Chronic myeloid leukemia (K562) cells, Cytochrome *c*, p53, Multicaspase, PCNA

## Abstract

**Background:**

Cancer is one of the most prominent human diseases which has enthused scientific and commercial interest in the discovery of newer anticancer agents from natural sources. Here we demonstrated the anticancer activity of ethanolic extract of aerial parts of *Pupalia lappacea* (L) Juss (Amaranthaceae) (EAPL) on Chronic Myeloid Leukemia K562 cells.

**Methods:**

Antiproliferative activity of EAPL was determined by MTT assay using carvacrol as a positive control. Induction of apoptosis was studied by annexin V, mitochondrial membrane potential, caspase activation and cell cycle analysis using flow cytometer and modulation in protein levels of p53, PCNA, Bax and Bcl2 ratio, cytochrome *c* and cleavage of PARP were studied by Western blot analysis. The standardization of the extract was performed through reverse phase-HPLC using Rutin as biomarker.

**Results:**

The results showed dose dependent decrease in growth of K562 cells with an IC_50_ of 40 ± 0.01 μg/ml by EAPL. Induction of apoptosis by EAPL was dose dependent with the activation of p53, inhibition of PCNA, decrease in Bcl2/Bax ratio, decrease in the mitochondrial membrane potential resulting in release of cytochrome *c*, activation of multicaspase and cleavage of PARP. Further HPLC standardization of EAPL showed presence 0.024% of Rutin.

**Conclusion:**

Present study significantly demonstrates anticancer activity of EAPL on Chronic Myeloid Leukemia (K562) cells which can lead to potential therapeutic agent in treating cancer. Rutin, a known anti cancer compound is being reported and quantified for the first time from EAPL.

## Background

Plant derived natural products gaining importance to cure various disease conditions. Side effects of several allopathic drugs and development of resistance to currently used drugs have led to increased emphasis on the use of plant materials as a source of medicines for a wide variety of human ailments. Incidentally plants and herbs are persistently being studied for the identification of novel therapeutic agents. Among the 2, 50,000 higher plant species on earth, more than 80,000 plants have medicinal values. India is one of the biodiversity centers with the presence of over 45,000 different plant species. Of these, about ~20,000 plants have good medicinal value. However, only ~7500 species are used for their medicinal values by traditional communities. It is well established that plants have always been useful source, for occurrence of anticancer compounds [1-3]. Approximately 60% of currently used anticancer chemotherapeutic drugs (vinblastin, vincristine) are derived from plant resource [4,5]. Moreover, traditionally, plants passed empirical testing against specific diseases and demonstrated that they are well tolerated in humans. Even though quite a few medicinal plants are applied against a wide variety of conditions, there are still numerous plants that have not been cross-tested in diseases apart from the traditional applications; one of such plant is *Pupalia lappacea*.

*Pupalia lappacea* (L) Juss. (Amaranthaceae) is an erect or straggling under shrub found in the hedges of fields and waste places from Kashmir to Kanyakumari and commonly known as forest Burr or creeping cock’s comb. In folklore medicine, the leaf paste of *Pupalia lappaceae* with edible oil is used to treat bone fractures and inflammatory conditions [6]. The fruit juice is applied locally for cuts, mixed with palm oil to treat boils and the fruit soup is used for cough and fever. In Africa, fruit is used as an ingredient in enema preparation; mixed with palm oil, it is applied as a dressing for boils and also applied to leprosy sores after making them bleed. Burnt plant is mixed with water to treat flatulence. Traditionally it is also used to treat jaundice, abdominal colics, cephalgias, diarrheas, paralysis, erectile dysfunction, vomiting and malaria [7]. Chemical investigations of *P*. *lappacea* revealed that foliage of this plant consists of 8 compounds, namely 1-docosanol, stearic acid, stigmasterol, sitosterol, N-benzoyl-L-Phenyl alaninol acetate, setosterol-3-O-D-glucopyranoside, stigmasterol-3-O-D-glucopyranoside and 20- hydroxyl ecdysone[8] The seeds are reported to consist of glycosides, saponins, steroids and alkaloids [9]. Aladedunye et al., reported the antioxidant activity of hexane and dichloromethane extract of *P*. *lappacea* foliage [8]. Sowemimo et al., in his preliminary studies reported the cytotoxic activity of whole plant of *P*. *lappacea* on HeLa cells [10]. Many of the natural antioxidants like curcumin, quercetin, resveratrol, berberine etc., are reported for potent anticancer activity in-vitro and in-vivo. Because of ethical considerations and the substantial time and expense required when using animal models, human cancer cell lines are preferred for most preliminary anticancer screening studies. The ability to inhibit cancer cell proliferation is considered as an indicator of anticancer potential, because the balance of tumor cell proliferation over cell death has been proposed to be one of the key factors in cancer evolution and progression. The present study was aimed to investigate *in vitro* anti proliferative activity of EAPL on K562 cells which is a proposed model for study of most of the cytotoxicity studies [11].

## Methods

### Plant material

The whole plant of *P*. *lappacea* was collected during flowering season from the Osmania University campus, Hyderabad, Andhra Pradesh, India, in the month of October 2010. Identification of plant was done by Dr. G. Bhagyanarayana, Taxonomist, Department of Botany, Osmania University, Hyderabad, India. The aerial parts (without flowers) were separated, cleaned, air dried and grounded to powder. The voucher specimen (PUL-203-07) is being maintained in department of Pharmacognosy, G. Pulla Reddy College of Pharmacy, Hyderabad, India.

### Preparation of plant extract

The dried powder of aerial parts (1000 g) was extracted with 80% aqueous ethyl alcohol (5 liters) at room temperature by maceration for 7 days. The extract was filtered and concentrated under reduced pressure in rotary flash evaporator. The concentrated organic extract was lyophilized to remove the moisture and traces of solvent. The final yield of aerial part was 1.85% (18.5 g). The lyophilized product was qualitatively tested for the presence of phytoconstituents by TLC and test tube reactions [12,13].

### Chemicals

3- (4, 5-dimethylthiazole-2-yl)-2, 5-diphenyl tetrazolium bromide (MTT), carvacrol were purchased from Sigma–Aldrich (Bangalore, India), Phosphate-buffered saline (PBS), RPMI medium, fetal bovine serum (FBS) were purchased from Gibco BRL (CA, USA). ECL reagent kit was purchased from GE Amersham whereas Nitrocellulose membrane from Millipore (Bangalore, India). Mouse monoclonal antibody against cytochrome *c* was from ChemiCon (CA, USA). Monoclonal antibodies of PARP (Poly (ADP-ribose) polymerase), BCl_2_ ((B-cell lymphoma 2) and Bax were procured from Upstate (Charlottesville, VA, and USA). All the other chemicals and reagents used were of analytical and molecular biology grade.

### Determination of effect of EAPL on cell proliferation by MTT assay

#### Cell culture

Human chronic myeloid leukemia K562 cells, Human embryonic kidney HEK-293 cells were procured from National Center for Cell Sciences, Pune, India. Cells were grown in RPMI media supplemented with 10% heat inactivated fetal bovine serum (FBS), 100 IU/ml penicillin, 100 mg/ml streptomycin and 2 mM-Glutamine. Cultures were maintained in a humidified atmosphere with 5% CO_2_ at 37°C. The cells were subcultured twice a week, seeding at a density of about 2*10^3^ cells/ml. cell viability was determined by the trypan blue dye exclusion method.

#### Cell proliferation assay

Cell viability was determined by MTT assay. 5 × 10^3^ cells/well (K562 and HEK-293) were seeded to 96-well culture plate and cultured with or without extract (10, 20, 30, 40, 50, 60, 70, 80, 90 & 100 μg/ml) or carvacrol (0, 0.5, 1, 10, 50,100 and 150 μg/ml) for 24 h in a final volume of 200 μl. After treatment, the medium was removed and 20 μl of MTT (5 mg/ml in PBS) was added to the fresh medium. After 3 h incubation at 37°C, cells were suspended in 100 μl of DMSO and plates were agitated for 1 min. Absorbance at 570 nm was recorded in a multi-well plate reader (Victor3 ^TM^, Perkin Elmer, USA). Percent inhibition of proliferation was calculated as a fraction of control (without extract).

### FACS analysis

#### Cell culture

1*10^4^ cells/well were seeded in 96 well plates and incubated for 2–3 hrs at 37°C in humidified 5% CO_2_ environment. Cells were treated with different concentrations (0, 25, 50, 100 μg/ml) of EAPL for 24 hrs, then harvested, stained and analyzed by Guava EasyCyte system for following experiments.

#### Annexin V assay

The Guava Nexin assay was conducted according to the manufacturer’s protocol. Briefly, cells after harvesting, resuspended in 50 μl complete RPMI medium. A volume of 150 μl staining solution (135 μl 1X apoptosis buffer, 10 μl Annexin V-PE, and 5 μl of 7-AAD) was then added to each well, incubated in the dark at room temperature for 20 min and acquired by using Guava EasyCyte system (5*10^3^ cells counted/sample, flow rate setting medium). The Nexin intensity gates were set to position the live population in the lower left corner of the dot plot. The angles of the gates were then positioned to divide the dot plot into four quadrants. Each quadrant of the dot plot contains a distinct population of cells that is dependent on the presence and intensity of cellular stains per cell.

#### Cell cycle analysis

Cell cycle analysis was conducted following the manufacturer’s instructions. After harvesting, cells were washed with 1X PBS twice, and then fixed overnight at −20°C in 70% ethanol. After fixing, stained for DNA content with propidium iodide, and acquired by using Guava EasyCyte system (5*10^3^cells counted/sample, flow rate setting medium). The cell cycle gates were set to a position in dot plot which corresponds to four markers showing marker 1: G1 phase, marker 2: S phase, marker 3: G2/M phase and marker 4: sub-G0/G1 cells in histogram.

#### Mitochondrial membrane potential analysis

The Guava MitoPotential assay was conducted according to manufacturer’s instructions. Briefly after harvesting, cells were resuspended in 200 μl of medium. A volume of 2 μl 100X JC-1 solution and 2 μl of 7-AAD was then added to each well. Plates were incubated for 30 minutes at 37°C in a 5% CO_2_ incubator and acquired on a Guava EasyCyte system (5*10^3^cells counted/sample, flow rate setting medium). The membrane mitopotential intensity gates were set to position the live population in the lower left corner of the dot plot. The angles of the gates were then positioned to divide the dot plot into four quadrants. Each quadrant of the dot plot contains a distinct population of cells that is dependent on the presences and intensity of cellular stains per cell. Finally percentage of apoptotic/dead cells corresponding to live cells was shown in dot plot.

#### Multicaspase activity analysis

Activation of multicaspase by EAPL was performed on Guava Easy Cyte Flow cytometer. Cells treated with EAPL were harvested, washed with PBS, stained with SR-VAD-FMK, 7-AAD and analyzed by flow cytometry according to manufacturer’s protocol (5*10^3^cells counted/sample, flow rate setting medium). The activation of multicaspase intensity gates were set to position the live population in the lower left corner of the dot plot. The angles of the gates were then positioned to divide the dot plot into four quadrants. Each quadrant of the dot plot contains a distinct population of cells that is dependent on the presence and intensity of cellular stains per cell.

### Western blot analysis

Western blot analysis was performed as previously described [14]. Briefly, cells were lysed in a lysis buffer and centrifuged (10,000 × *g*) for 10 min. The protein content of the supernatant was determined according to the Bradford method [15] and used as the whole-cell extracts. Proteins (100 μg) were separated on 8–12% sodium dodecyl sulphate - polyacrylamide gels (SDS-PAGE) along with protein molecular weight standards and electrophoretically transferred to nitrocellulose membrane (Bio-Rad Laboratories, Hercules, CA). Non specific binding sites on the membranes were blocked with 5% (w/v) nonfat dry milk after checking the transfer using 0.5% Ponceau *S* in 1% acetic acid and then probed with a relevant antibody (Bax, Bcl2, PARP at 1:1000 dilution) for 8–12 h at 4°C followed by detection using peroxidase-conjugated secondary antibodies and Super Signal West Pico Chemiluminescence Substrate (Thermofisher scientific, USA). Equal protein loading was detected by probing the membrane with β-actin antibodies.

Release of cytochrome *c* from mitochondria was measured by immunoblot assay as previously described [16] with some modifications. Briefly, cells were washed once with ice-cold PBS and gently lysed for 30 s in 80 μl ice-cold lysis buffer (250 mM sucrose, 1 mM EDTA (ethylene diamine tetra acetic acid), 0.05% digitonin, 25 mM Tris, pH 6.8, 1 mM dithiothrietol, 1 mg/ml aprotinin, 1 mg/ml pepstatin, 1 mg/ml leupeptin, 1 mM PMSF (phenyl methane sulfonyl fluoride) and 1 mM benzamidine). Lysates were centrifuged at 12,000 × *g* at 4°C for 5 min to obtain the extracts (cytosolic extracts free of mitochondria). Supernatants were electrophoresed on 8-15% SDS polyacrylamide gel and then analyzed by Western blot using cytochrome *c* antibody.

### HPLC standardization

The sample was analyzed by reverse phase-high performance liquid chromatography using Shimadzu class LC-20 AD HPLC system, composed by a binary pump, 100 μl injection loop, Photo Diode Array (PDA) detector set at 250–350 nm under room temperature with a flow rate of 0.9 ml/min. Phenomenex Luna 5_ C-18 (2) (150 mm × 4.6 mm) was used as stationary phase. The mobile phase constituted 0.5% formic acid and acetonitrile (70:30%, v/v). A serial dilution of standard rutin resulting to 15 ppm, 25 ppm, 50 ppm, 75 ppm and 100 ppm solutions were used for preparing calibration curve (Concentration Vs Area Under Curve). The amount of rutin present in EAPL was quantified from the standard graph.

### Statistical analysis

All experiments were carried out in triplicate. Data were expressed as means ± SEM. Differences were evaluated by one-way analysis of variance (ANOVA) test completed by Dennett’s test. * denotes p < 0.05, ** denotes p < 0.01 and *** denotes p < 0.001. The 50% inhibitory concentration (IC_50_) was calculated by nonlinear regression curve with the use of Graphpad Prism version 5.0 for Windows (Graphpad Prism Software, San Diego, CA, USA).

## Results and discussion

### Phytochemical analysis

The aqueous ethanolic extract was found to contain steroids, terpenoids, flavonoids and/or their glycosides, tannins and carbohydrates. The alkaloids, coumarins, cardiac glycosides were absent.

### The determination of IC_50_ by MTT assay

**Figure 1:**
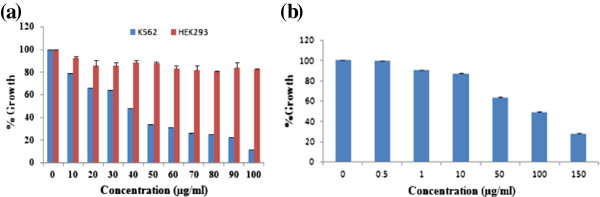
**MTT assay: Cells treated with EAPL (a), Carvacrol for 24 h in K562 cell lines (b).** Values were expressed as mean ± SEM (P < 0.05).

In an effort to gain mechanistic insights of EAPL-induced apoptosis on K562 cells, the anti-proliferative activity was evaluated by MTT assay along with normal (HEK 293) cells. As shown in Figure 1a, a dose-dependent decrease in the growth of cells was observed with increasing concentrations of EAPL and no significant difference was observed in the growth of normal cells. IC_50_ value of EAPL on K562 cells was calculated to be 40 ± 0.01 μg/ml (mean ± SEM) at 24 h by using Graphpad Prism. As a positive control, carvacrol was included in the study and the IC_50_ was determined to be was 110 μg/ml (Figure 1b) as reported earlier [17]. The assay suggests that EAPL is more specific towards cancer cells and ~3 fold more effective compare to carvacrol.

### Morphological observations

**Figure 2:**
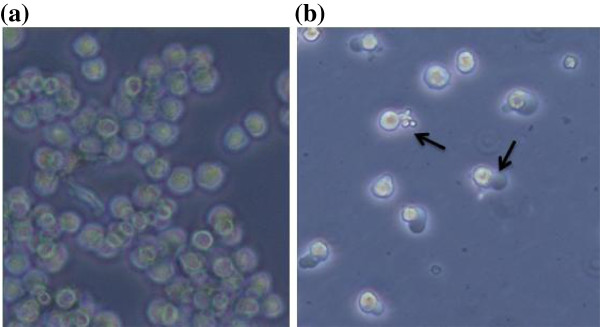
**Microscopic photographs: untreated (a), treated (100 μg/ml) (b) cells.** Arrows indicate apoptotic cells.

In order to evaluate the cause of growth inhibition of K562 cells by EAPL, characteristic features of apoptosis were studied. Apoptosis or programmed cell death is recognized by characteristic pattern of morphological, biochemical, and molecular changes occurring in a cell [18]. EAPL treated (100 μg/ml) cells for 24 h showed prominent morphological changes resembling cell shrinkage with rounding of cells and formation of membrane blebs (Figure 2b) compare to control (Figure 2a) cells which is a characteristic feature of apoptosis as evidenced by microscopic studies.

### Annexin V assay

**Figure 3:**
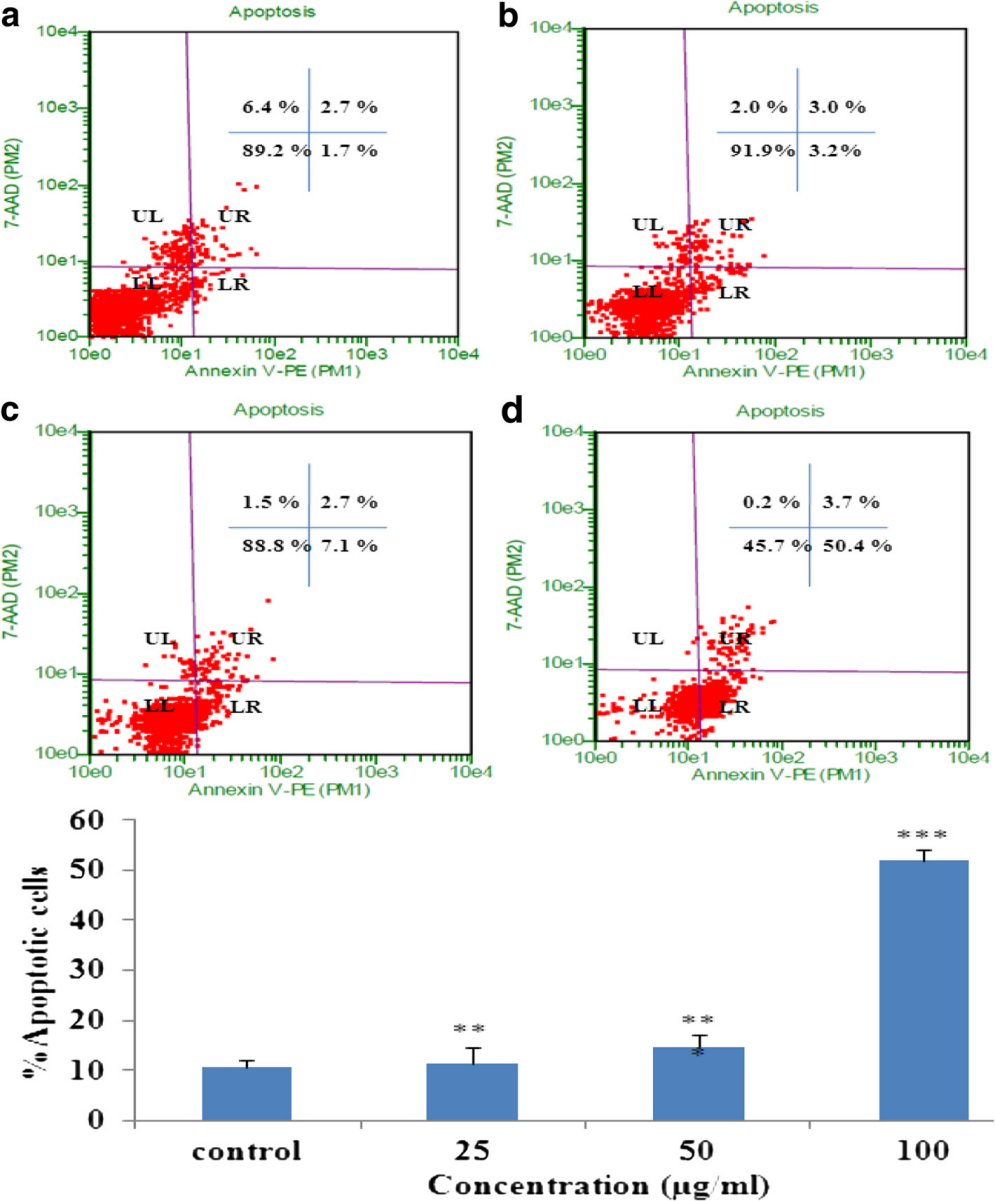
**Annexin V assay: 5 x 10**^**3**^**cells were treated with EAPL at different concentrations for 24 h analyzed by flow cytometer.** (**a**) Control (**b**) 25 μg/ml (**c**) 50 μg/ml (**d**) 100 μg/ml. UL-Dead cells. UR-Late apoptotic cells. LR-Early apoptotic cells LL-live cells. Bar graph represents the % apoptotic cells (mean ± SEM).

Redistribution of phosphatidylserine to the external side of the cell membrane occurs due to perturbation in the cellular membrane, is one of the biochemical features of apoptosis [19]. Annexin V, a recombinant phosphatidylserine-binding protein, interacts strongly and specifically with phosphatidylserine residues and is used for the detection of apoptosis [20]. Staining of cells with 7-AAD discriminate early apoptotic and dead cells. From Figure 3, the vast majority of K562 cells in the untreated (3a) were healthy and thus unstained for annexin V and 7-AAD. Whereas cells treatment with EAPL resulted in dose dependent increase in the Annexin V and 7-AAD positive cells (3b, 3c, 3d) demonstrating induction of apoptosis.

### Cell cycle analysis

**Figure 4:**
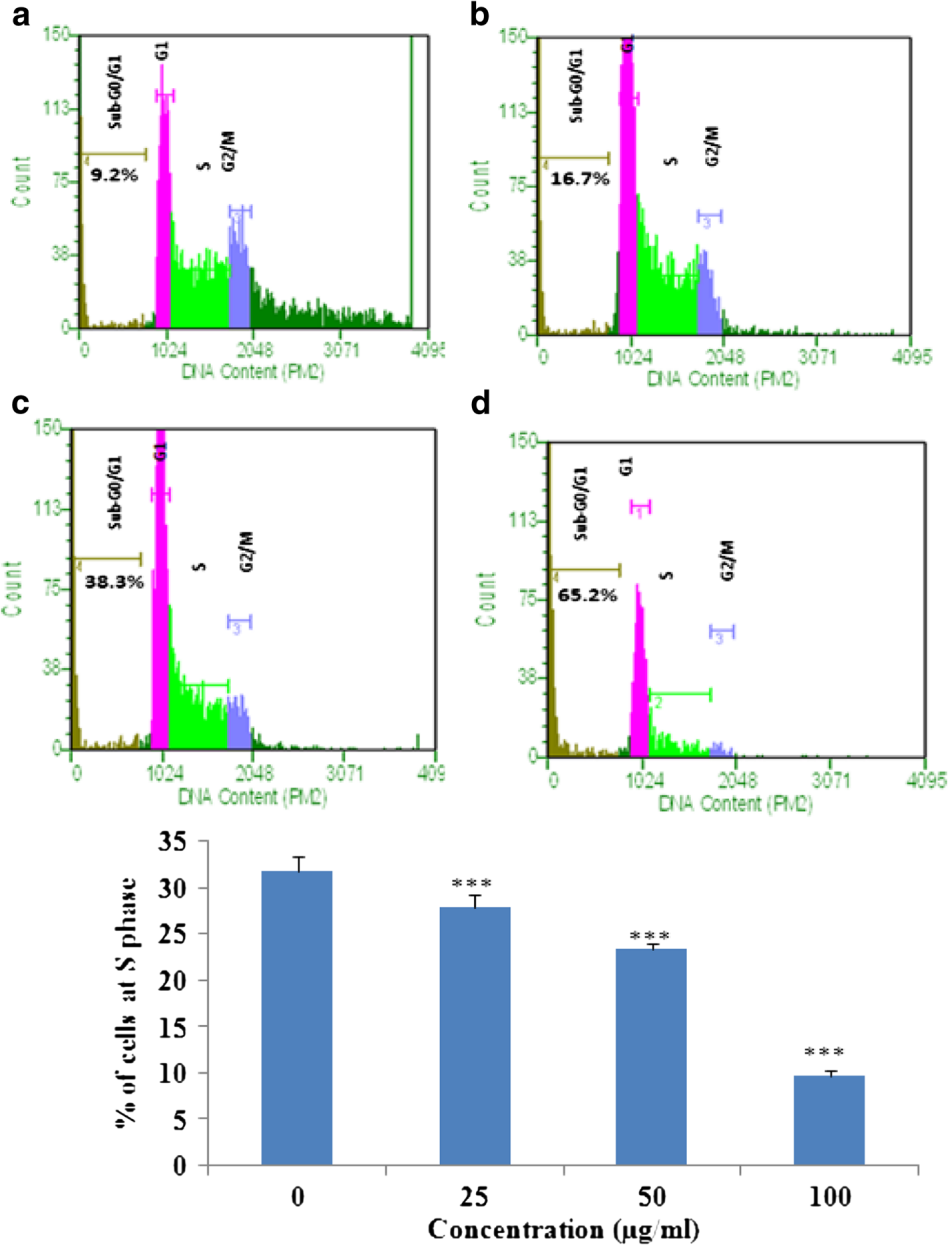
**Cell cycle analysis: Cells exposed to different concentrations of EAPL (25,50 and 100 μg/ml) for 24 h.** (**a**) Control (**b**) 25 μg/ml (**c**) 50 μg/ml (**d**) 100 μg/ml. Bar graph represents the % cells at S phase (mean ± SEM).

Loss of DNA content is a typical characteristic feature of apoptosis. Staining of cells with propidium iodide (PI) and analyzing by flow cytometer would helps in evaluating the cells at different (subG0/G1, G1, S, G2/M) phases of cell cycle. K562 cells were exposed with different concentrations (25, 50, 100 μg/ml) of EAPL for 24 h, washed and harvested and then analyzed in flow cytometry (FACS). The percentage of cells in each phase of the cell cycle was calculated and shown in Figure 4. A dose dependent increase of sub G0/G1 phase (apoptotic peak) and decrease of S phase was observed, indicating the induction of apoptosis and inhibition of DNA synthesis in S phase. These results are in accordance with the previous results [14].

### p53 mediated apoptosis

**Figure 5:**
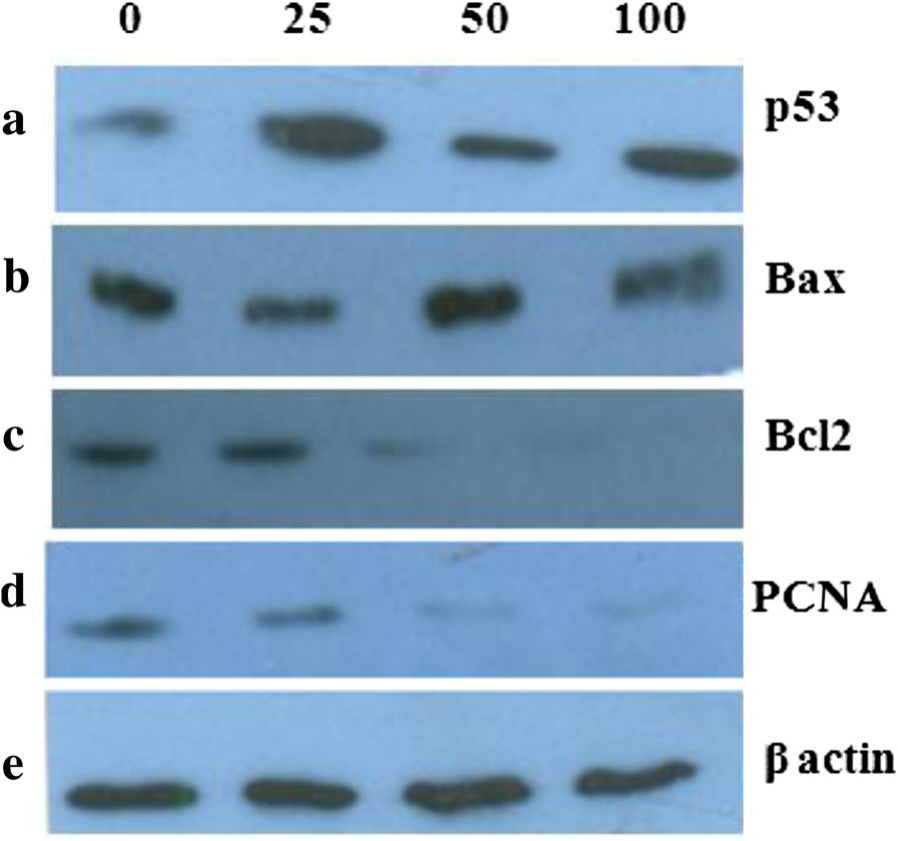
**Immunoblot analysis: Lane 1:0 μg;lane 2:25 μg;lane 3:50 μg;lane 4:100 μg.** Equal loading confirmed by β-actin.

Further to understand the underlying molecular mechanism of apoptosis induced by EAPL, activation of the p53-mediated signaling network was evaluated. Modulation of p53 levels was evaluated by Western blot analysis using p53 antibody in K562 cells treated with different concentrations (25, 50, 100 μg/ml). The results showed an increase in p53 levels in a dose dependent manner (Figure 5a). Bax homodimers are inducers of apoptosis whereas Bax-Bcl2 heterodimer formation results in cell survival. Both Bax and Bcl2 are transcriptional targets of p53 [21,22]. The levels of the pro-apoptotic protein Bax and anti-apoptotic protein Bcl2 were measured using Western blot. There is a significant decrease in the levels of Bcl2 (Figure 5c) with a little or no alteration in Bax protein, indicating the formation of Bax homodimers (Figure 5b). These changes resulted in increase within the Bax/Bcl2 ratio thereby pushing the cellular balance in the direction of apoptosis. Subsequently, proliferating cell nuclear antigen (PCNA), the marker of dividing cells, specifically inhibited by p53 [23,24] was also studied. The results illustrate a dose-dependent decrease in the expression of PCNA, suggesting induction of apoptosis (Figure 5d).

### MitoPotential and multicaspase activation assay

**Figure 6:**
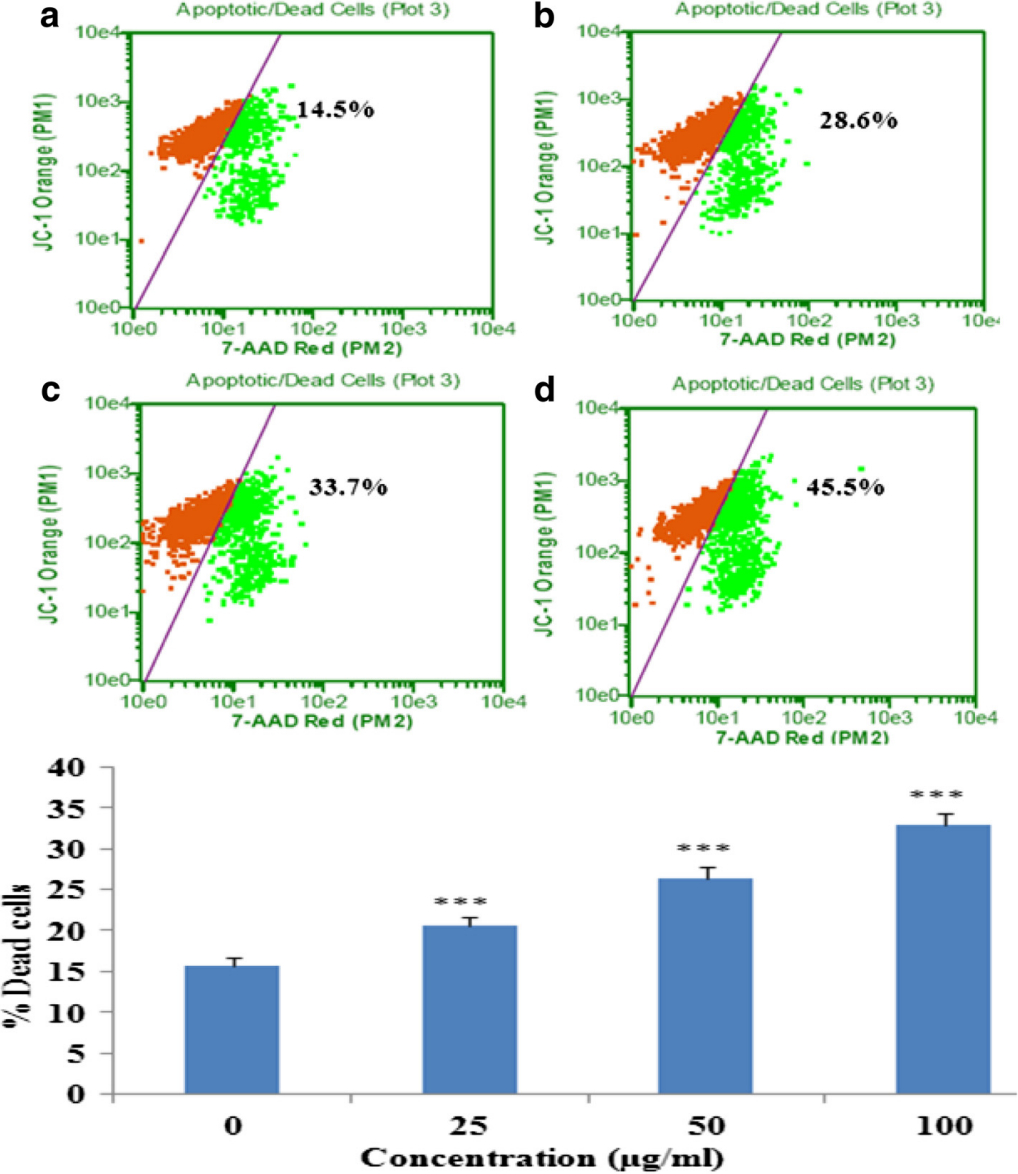
**MitoPotential analysis.** K562 cells treated with (25, 50, and 100 μg/ml) or without EAPL for 24 h and analyzed by flow cytometer. D-Dead cells. (**a**) Control (**b**) 25 μg/ml (**c**) 50 μg/ml (**d**) 100 μg/ml. Bar graph represents the % dead/apoptotic cells (mean ± SEM).

**Figure 7:**
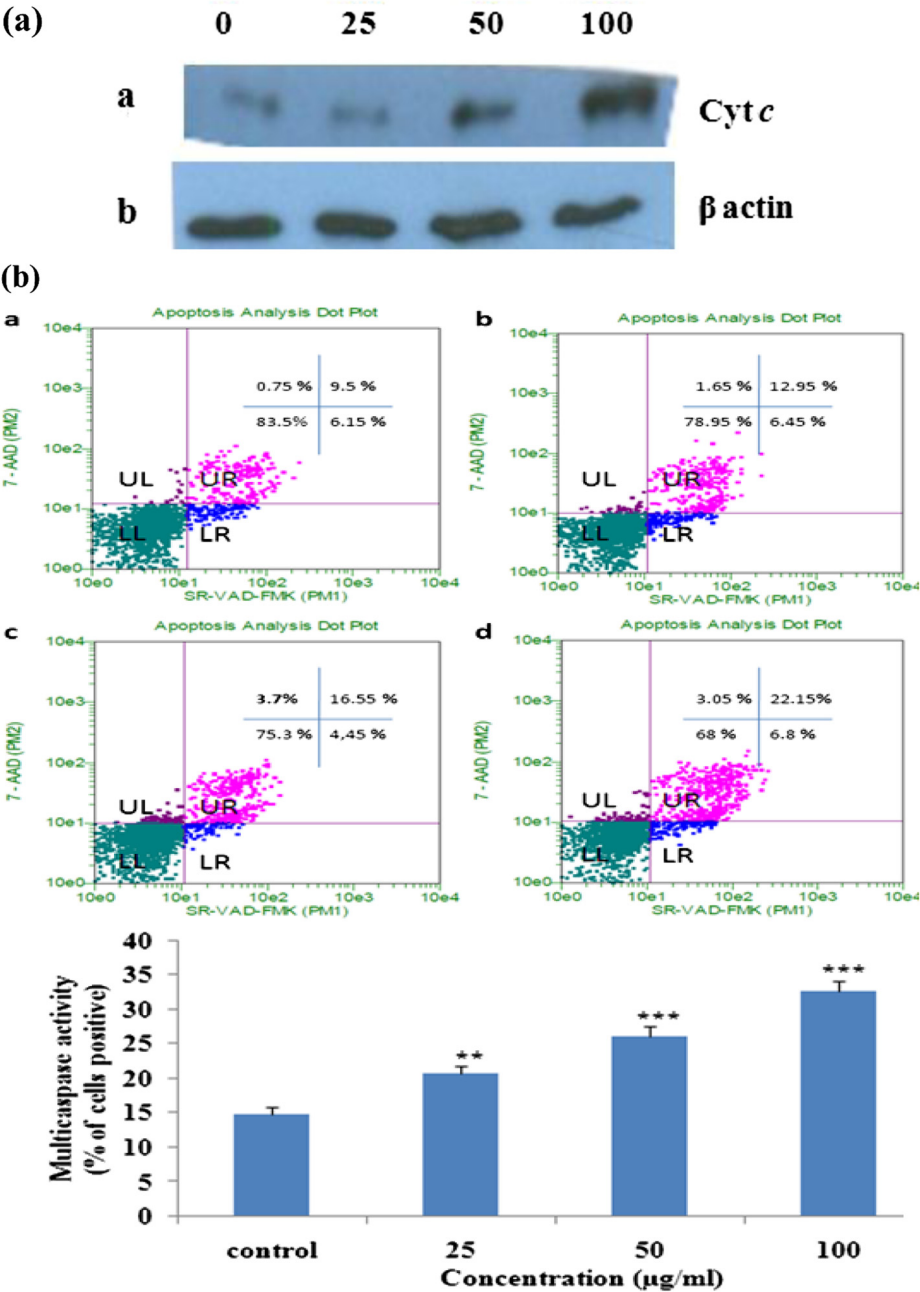
**(a) Immunoblot analysis of Cyt *c *expression in EAPL treated (25, 50, 100 μg**/**ml)K562 cells.** Equal loading confirmed by β-actin. (**b**) Multicaspase analysis. K562 cells treated with (25, 50, and 100 μg/ml) or without EAPL for 24 h and analyzed by flow cytometer. (**a**) Control (**b**) 25 μg/ml (**c**) 50 μg/ml (**d**) 100 μg/ml. Bar graph represents the % of caspase active cells (mean ± SEM).

Loss of mitochondrial inner transmembrane potential (ΔΨm) is often [25-28], but not always [29,30] observed to be associated with the early stages of apoptosis. Collapse of this potential is believed to coincide with the opening of the mitochondrial permeability transition pores, leading to the release of cytochrome *c* into the cytosol. In cytoplasm, cytochrome *c* combines with caspase-9, Apaf-1 and dATP to form the apoptosome complex [31] which in turn activates caspase- 9, -3 and −7. The alteration in membrane mitopotential of the apoptotic cells was determined by flow cytometry and the results showed a dose dependent decrease in the membrane potential (Figure 6). Further the release of cytochrome *c* from mitochondria into cytosol was analyzed by Western blot analysis in cytosolic fractions of EAPL treated cells. There is a dose dependent increase in the levels of cytochrome *c* in the cytoplasm indicating the execution of apoptosis (Figure 7a). In addition activation of multicaspases was determined by FACS analysis and the results clearly demonstrated an increase in multicaspases activation with increase in concentration of EAPL (Figure 7b).

### Cleavage of PARP

**Figure 8:**
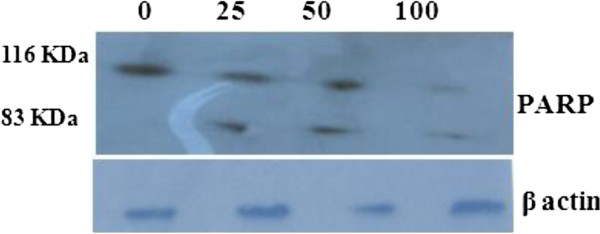
**Immunoblot analysis of PARP cleavage in EAPL treated (25, 50, 100 μg/ml) K562 cells.** Equal loading confirmed by β-actin.

PARP (poly (ADP-ribose) polymerase) catalyzes the poly ADP-ribosylation of a variety of nuclear proteins with NAD as substrate. Upon DNA damage, PARP gets activated and depletes NAD as well as ATP in an attempt to repair the broken DNA. During apoptosis, caspase-3 inactivates PARP by cleaving it into 83 and 24 kDa fragments and thereby preserves ATP resources of the cell for apoptosis [32,33]. Results from immunoblot analysis using antibody that recognizes uncleaved (116 kDa) and cleaved (83 kDa) fragments of PARP, clearly demonstrate dose dependent inactivation of PARP in EAPL treated cells (Figure 8). The above mentioned studies clearly demonstrate that EAPL induces apoptosis in K562 cells by intrinsic death pathway.

### HPLC standardization

Under the set of given analytical conditions, the retention time of standard rutin was observed as 3.8 min (see Additional file 1: Figure S1). The retention time of Rutin present in the sample was found to be identical (see Additional file 1: Figure S1) and the amount of Rutin in EAPL was calculated to be 0.024 % (w/w).

## Conclusion

In conclusion, the current work demonstrates the antiproliferative effect of EAPL to induce programmed cell death in chronic myeloid leukemia (K562) cells with an IC_50_ of 40 ± 0.01 μg/ml, showing its anticancer property. It also explains the underlying molecular proceedings occurring in the presence of EAPL. Together, morphological changes such as cell shrinkage, rounding of cells, membrane blebbing, activation of p53, cell cycle arrest at S phase, increase in annexin V positive cells, increase in Bax/Bcl2 ratio, decrease in mitochondrial membrane potential and increase in cytochrome *c* release from mitochondria, increase in caspase activity, cleavage of PARP and inhibition of PCNA demonstrate the molecular mechanism of programmed cell death by EAPL. Many of the natural antioxidants have been reported for anti cancer activity by the mechanism not yet clear but one example is curcumin induces apoptosis via a ROS-associated mechanism that converges on JNK activation, and to a lesser extent via a parallel ceramide-associated pathway [34]. Thus, the current work clearly reveals that EAPL could be a potent anti-cancer agent against chronic myeloid leukemia. Study of individual phytoconstituents of the EAPL extract may form the basis for future studies.

The use of natural drugs or food products rich in antioxidant property has been drawn great attention by many scientists for chemotherapeutic purposes, in part because these foods are generally recognized as safe. But natural drugs and food components showing strong antioxidant potential cannot always result in anticancer activities since the biological process of cancer development may be affected by phytochemicals via different mechanisms in addition to antioxidant effects. Many cell-culture based studies have been performed in an attempt to elucidate the relationship between antioxidant activities and anticancer properties of some plant-based drugs. To this end, conflicting findings were observed. Even though mechanism of antitumor activity of most of the plant based drugs has been established, still it is unclear with respect to mechanism of cancer cell death based on antioxidant property [35]. Many studies propose that there are additional functions accountable for the antitumor activity produced by these plant-based drugs that go beyond their antioxidant ability. Extensive molecular and metabolic studies are required to discover these other pathways [36]. Beside the use of cell-culture models, animal experiments and human clinical trials should be employed to explore the possible applications of phytochemical antioxidant rich drugs. These studies will provide more physiological insight into whether antioxidant capacities of plant base drugs are directly linked to their anticancer activities.

## Competing interests

The authors declare that there is no conflict of interest that could influence the impartiality of the research reported. This article is original and has been written by the stated authors who are all aware of its content and approve its submission. It has not been published previously or under consideration for publication elsewhere, no conflict of interest exists. If accepted, the article will not be published elsewhere in the same form, in any language, without the written consent of the publisher.

## Authors' contributions

AR, AM participated in the design of the study, performed most of the experimental work and drafted the manuscript. VS performed extraction and phytochemical analysis, selection of the journal. AMK, VKI, AR, AM performed western blot analysis. BMR involved in review of the paper. All authors read and approved the final version of the manuscript.

## Supplementary Material

Additional file 1 **Figure S1A:** HPLC chromatogram of standard Rutin (15, 25, 50, 75 and 100 ppm) and sample (EAPL). **Figure S1B**: HPLC chromatogram of ethanolic extract of aerial parts of Pupalia lappacea. (DOC 66 kb) Click here for file
