# Supplementary material for: Anticancer activity of Pupalia lappacea on chronic myeloid leukemia K562 cells
Source: Daru. 2012 Dec 5;20(1):86. doi: 10.1186/2008-2231-20-86 (PMC3559012; doi:10.1186/2008-2231-20-86)
Supplement: Additional file 1 — Figure S1A: HPLC chromatogram of standard Rutin (15, 25, 50, 75 and 100 ppm) and sample (EAPL). Figure S1B: HPLC chromatogram of ethanolic extract of aerial parts of Pupalia lappacea. (DOC 66 kb) [file 2008-2231-20-86-S1.doc]

**HPLC standardization:**

The sample was analyzed with Reverse phase High Performance Liquid Chromatography carried out on Shimadzu class LC-20AD HPLC system, composed by a binary pump, 100 µl injection loop, Photo Diode Array (PDA) detector set at 250-350 nm under room temperature with a flow rate of 1ml/min. The stationary phase used was Phenomenex Luna 5µ C-18 (2) (150 mm x 4.6 mm) and mobile phase 5 % Glacial acetic acid in water and acetonitrile (80:20 % v/v). A serial dilution of standard Rutin resulting to 15 µg/ml, 25 µg/ml, 50 µg/ml, 75 µg/ml and 100 µg/ml solutions were used for preparing calibration curve (Concentration Vs Area Under Curve). The amount of rutin present in the ethanolic extract of aerial parts of *Pupalia lappacea* was quantified from the standard graph.

Under the set of given analytical conditions, the retention time of standard Rutin was observed as 3.8 min (Fig 1A). The retention time of Rutin present in the sample was found to be identical (Fig 1B) and the amount of Rutin in ethanolic extract of aerial parts of *Pupalia lappacea* was calculated to be 0.024 % w/w.

**Figure 1A**

**
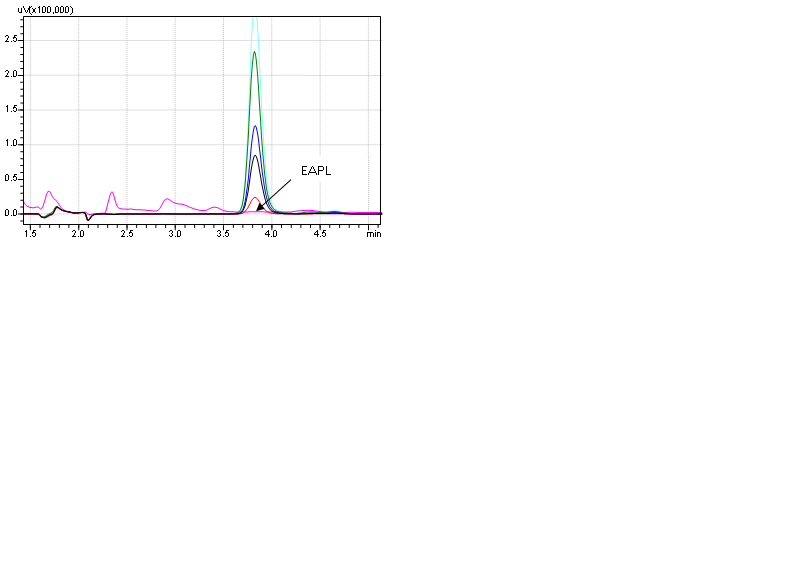
**

**Fig 1A:** HPLC chromatogram of standard Rutin (15, 25, 50, 75 and 100 ppm) and sample (EAPL).

**Figure 1B**

**
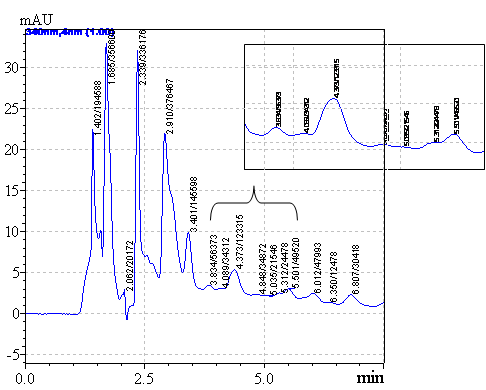
**

**Fig 1B:** HPLC chromatogram of ethanolic extract of aerial parts of *Pupalia lappacea.*
